# The Pioneer Transcription Factor Foxa2 Modulates T Helper Differentiation to Reduce Mouse Allergic Airway Disease

**DOI:** 10.3389/fimmu.2022.890781

**Published:** 2022-08-08

**Authors:** Diana C. Yánez, Ching-In Lau, Eleftheria Papaioannou, Mira M. Chawda, Jasmine Rowell, Susan Ross, Anna Furmanski, Tessa Crompton

**Affiliations:** ^1^ UCL Great Ormond Street Institute of Child Health, London, United Kingdom; ^2^ School of Medicine, Universidad San Francisco de Quito, Quito, Ecuador; ^3^ School of Life Sciences, University of Bedfordshire, Luton, United Kingdom

**Keywords:** Foxa2, Shh, Th2 airway inflammation, Th1, Th2

## Abstract

Foxa2, a member of the Forkhead box (Fox) family of transcription factors, plays an important role in the regulation of lung function and lung tissue homeostasis. FOXA2 expression is reduced in the lung and airways epithelium of asthmatic patients and in mice absence of Foxa2 from the lung epithelium contributes to airway inflammation and goblet cell hyperplasia. Here we demonstrate a novel role for Foxa2 in the regulation of T helper differentiation and investigate its impact on lung inflammation. Conditional deletion of *Foxa2* from T-cells led to increased Th2 cytokine secretion and differentiation, but decreased Th1 differentiation and IFN-γ expression *in vitro*. Induction of mouse allergic airway inflammation resulted in more severe disease in the conditional Foxa2 knockout than in control mice, with increased cellular infiltration to the lung, characterized by the recruitment of eosinophils and basophils, increased mucus production and increased production of Th2 cytokines and serum IgE. Thus, these experiments suggest that Foxa2 expression in T-cells is required to protect against the Th2 inflammatory response in allergic airway inflammation and that Foxa2 is important in T-cells to maintain the balance of effector cell differentiation and function in the lung.

## Introduction

Allergic asthma is a common genetically complex inflammatory lung and airways disease in which T helper 2 (Th2) responses and inflammation are triggered by inhalation of allergens from the environment (1, 2). Asthma involves abnormal interactions between multiple cell types. Epithelial cells are the source of excess airway mucus, and they are also believed to initiate the response to the allergen, by secretion of cytokines and other intercellular signalling molecules, leading to recruitment of immune and inflammatory cells. Sensitization to the allergen then drives differentiation of naïve CD4 T-cells to the Th2 effector subset, which further promote allergic asthma by secretion of cytokines and chemokines, amplifying epithelial cell, eosinophil, mast cell and basophil responses, leading to IgE production, chronic inflammation and airway remodelling (3, 4). The ways in which inhalation of the allergen lead to sensitization and CD4 Th2 differentiation in susceptible individuals are still unclear, and susceptibility is likely to be influenced by a combination of factors that affect barrier function; factors that affect epithelial cell homeostasis, function and cytokine secretion; and T-cell intrinsic factors that predispose their differentiation to Th2.

Foxa2 belongs to the highly conserved Foxa subfamily of Forkhead transcription factors. It is an essential regulator of development, differentiation and cell identity in many tissues both in the embryo and adult (5). Foxa2 plays an important role in lung development and function, and its mutation is associated with lung cancer and lung Th2 inflammation (6–13). Conditional deletion of Foxa2 from murine respiratory epithelial cells causes alveolar malformation, airspace enlargement, goblet cell hyperplasia, mucus overproduction, and spontaneous Th2 inflammation with Th2 cytokine and chemokine secretion (7, 14). Suppression of FOXA2 expression in the lung epithelia is also associated with Th2-mediated disease, goblet cell hyperplasia and mucus production: *FOXA2* expression was found to be lower than normal in the airways of mild/moderate asthmatic patients and its expression was negatively correlated with expression of Mucin genes, whereas constitutive expression of Foxa2 in murine lung epithelium reduced mucus metaplasia in an asthma model (12). Foxa2 is believed to suppress mucus production by counteracting the effects of IL-13/STAT6 signalling, which *via* their downstream effector SPDEF increase expression of the mucin gene *Muc5ac*. Foxa2 has been shown to suppress *Spdef* expression, whereas IL-13 *via* SPDEF can suppress *Foxa2* expression, indicating opposing roles for Foxa2 and IL-13 signalling in lung epithelial cell homeostasis and inflammation (15–17). In support of this, conditional deletion of Foxa2 from respiratory epithelium cells during postnatal lung development increased the production and release of leukotrienes and Th2 cytokines IL-13 and IL-4 in the lung (18).

Foxa2 is also associated with the Hedgehog (Hh) signalling pathway, but it interacts with Hh signalling in different tissues in different ways: it can be a direct target gene of Sonic Hedgehog (Shh) signalling; it can function to maintain Shh expression; or it can modulate expression of components of the Shh signalling pathway (19–21). In lung, conditional deletion of both Foxa1 and Foxa2 from lung epithelial cells reduced expression of Shh, but Shh-/- lung showed normal levels of expression of Foxa1 and Foxa2, suggesting that Foxa1/2 are required for Shh expression but are not direct targets of Shh signalling in lung epithelial cells (6). Many studies have shown that Shh upregulation in lung epithelial cells exacerbates allergic airway inflammation (22–28). Additionally, Shh signalling to naïve CD4 T-cells promotes their differentiation to Th2 *in vitro* and drives allergic airway disease (AAD) in lung *in vivo* (23, 24, 29, 30).

Little is known about the function of Foxa2 in the immune system. Foxa2 is expressed in thymic epithelial cells (TEC) and conditional deletion of both Foxa1 and Foxa2 from TEC altered their function, resulting in a smaller thymus with fewer conventional CD4 T-cells but more CD4 T regulatory (Treg) cells (31). Foxa2 is also expressed in developing T-cells in the thymus, and *Foxa2* is a transcriptional target of Shh signalling during early T-cell development (32, 33). However, conditional deletion of Foxa2 from developing T-cells using transgenic Cre under control of the CD4 promoter resulted in efficient excision but grossly normal T-cell development, with normal thymocyte numbers, and a modest reduction in the proportion of peripheral CD4 T-cells (33).

Given the functional relationship between Foxa2 and Shh, the opposing roles of Shh and Foxa2 in allergic asthma, the role of Hh in driving Th2 differentiation, and the importance of CD4+ Th2 responses in amplifying asthma, we investigated the function of Foxa2 in CD4 T-cells in AAD. We show that conditional deletion of Foxa2 from T-cells exacerbated Th2 inflammation on induction of AAD *in vivo* and inhibited the differentiation of CD4 T-cells to Th1 *in vitro*, but increased their differentiation to Th2.

## Materials and Methods

### Mice

Foxa2^flox/flox^ mice provided by S-L Ang (34); CD4-Cre-trangenic (purchased from Jackson Laboratory); and wild type (WT) (C57BL/6) mice were bred and maintained on C57BL/6 background at University College London and crossed to generate Foxa2^flox/flox^ CD4cre+ mice (referred to as Foxa2cKO) and littermate Foxa2^flox/flox^ CD4cre^neg^ (referred to as control) (33). Mouse studies were approved by the British Home Office.

### Papain-Allergen Challenge Model and Cell Isolation

To induce AAD, 6 to 8- week-old mice were administered papain protease (papain) (Sigma) (**Figures S1**, **2**). In brief, on day 0 and 7 mice were administered 25μg papain in 25μl PBS or control 25μl PBS intranasally under anaesthesia drop by drop onto the nostril to introduce papain into the airways and lung as mice inhale. At day 10, mice were sacrificed and bronchoalveolar lavage (BAL), lungs and mediastinal lymph nodes (mLN) were analysed by flow cytometry using gating strategies and markers shown in **Figure S1** (24, 35, 36). This treatment protocol induced AAD in C57BL/6 mice, with increased immune and inflammatory cell infiltration to BAL and lung; increased total serum IgE; and IL-4 and IL-13 cytokines in BAL and lung; compared to PBS-treated controls (**Figures S1**, **2**).

### Cell Culture

CD4 T-cells from spleen, purified with EasySep™ Mouse CD4 T-Cell Isolation Kit (StemCell Technologies) were cultured at 1×10^6^ cells/ml in complete RPMI (RPMI+10%FBS+5%Pen/Strep) and anti-CD28 (1μg/ml) in 96-well plates, coated with anti-CD3ϵ ((5 μg/ml) at 37°C, 5% CO_2_ for 2h), with addition of for: Th1 anti-IL-4 (5μg/ml), rmIL-12 (10ng/ml); Th2 anti-IL-12 (5μg/ml), anti-IFN-γ (5μg/ml), rmIL-4 (20ng/ml); (Th0, no additions); using cytokines and antibodies listed in **Table S1**. In some experiments, cells were restimulated for 16 hours with soluble anti-CD3/anti-CD28 (1μg/ml).

### Flow Cytometry and Cell Sorting

Cells were stained as described (37) using antibodies listed (**Table S1**). Data acquired on a C6 Accuri (BD Biosciences) or LSRII (BD Biosciences) were analysed using FlowJo v10 (Tree Star). Debris were excluded according to FSC/SSC profiles and singlets were selected by gating on FSC-H/FSC-A (**Figure S1**). Staining against intracellular (ic) cytokines followed 4hour stimulation at 37°C with PMA (50 ng/mL, Sigma), Ionomycin (500ng/mL, Sigma), and BrefeldinA (eBioscience). Transcription factor staining was as described (37), using isotype-matched antibodies as negative controls.

For cell sorting, digested lung suspensions stained with Dapi (to exclude dead cells), and anti-CD45, anti-CD326, anti-CD4, anti-CD8, anti-CD11c and anti-CD3 were sorted using FACS AriaIII (BD Biosciences) for epithelial cells (CD45-CD326+), CD4 T-cells (CD3+CD4+), CD8 T-cells (CD3+CD8+) and CD4+ dendritic cells (CD4+CD11c+).

### Quantitative Reverse Transcriptase Polymerase Chain Reaction (QRT-PCR)

RNA was extracted from whole homogenised lungs using Absolutely RNA miniprep kit (Agilent), and from purified CD4 T-cells and FACS-sorted cells using PicoPure kit (Applied Biosystems, USA). cDNA synthesized using High-Capacity cDNA reverse transcription kit (Applied Biosystems) following manufacturer’s guidelines, was analysed on an iCycler (Bio-Rad). Transcripts were measured relative to the housekeeping gene *Hprt* as described (38). Primers were purchased from Qiagen, Venlo, Netherlands.

### Enzyme-Linked Immunosorbent Assay

ELISAs for IgE, IL-13 and IL-4 used Ready-Set-Go! Kits (eBioscience) and for Shh Mouse Shh ELISA Kit (R&D Systems), according to manufacturers’ instructions.

### Histology

Lungs were formalin-fixed, paraffin-embedded, and sectioned for H&E and Periodic Acid Schiff (PAS) staining, photographed using Zeiss AxioCam digital camera, Axioplan (NDU) microscope and AxioVisionv4.8 software, and analysed using ImageJ. Semiquantitative H&E assessment was performed blind twice by independent observers to score for cellular infiltration: 0–1, minimal; 1–2, moderate; 2–3, severe. PAS staining, using PAS Stain Kit (Abcam) following manufacturer’s protocol, was assessed blind for mucus production as: 0–1, minimal; 1–2, moderate; 2–3, severe.

### Statistics

Unpaired two-tailed Student’s t-tests were used and probabilities considered significant if p<0.05(*), p< 0.01(**), p< 0.001(***), p< 0.0001(****).

## Results

### Conditional Deletion of Foxa2 From T-Cells Increases Inflammatory Cell Infiltration in a Murine Model of Allergic Airway Inflammation

To investigate Foxa2 function in T-cells on induction of AAD, we used Foxa2cKO mice in which floxed alleles of *Foxa2* were conditionally deleted from T-cells using CD4-Cre (Foxa2cKO). Conditional deletion of Foxa2 from T-cells was successful, and *Foxa2* expression was below detection in FACS-sorted lung CD4 and CD8 T-cells. As expected, *Foxa2* was expressed at normal levels in FACS-sorted lung epithelial cells (CD45-CD326+). Deletion was incomplete in the lung CD11c+CD4+ dendritic cell population, in which expression was reduced by ~3-fold. Comparison between control populations showed that Foxa2 is ~6-fold more highly expressed in lung epithelial cells than in lung T-cells (**Figure S3A**). To induce AAD, we used papain as allergen, which induces allergic airway inflammation by initiating a Th2 immune response and is linked to occupational and non-occupational asthma and allergy in humans (29, 35, 39–45). We administered intranasal papain in PBS to Foxa2cKO (Foxa2f/fCD4Cre+) and control (Foxa2f/fCD4Cre^neg^) mice on day 0 and day 7 and sacrificed mice on day 10, treating with PBS alone at the same time points as negative control (**Figure S1A**). This protocol of intranasal papain administration has previously been shown to induce allergic airway inflammation in mice (35).

**Figure 1:**
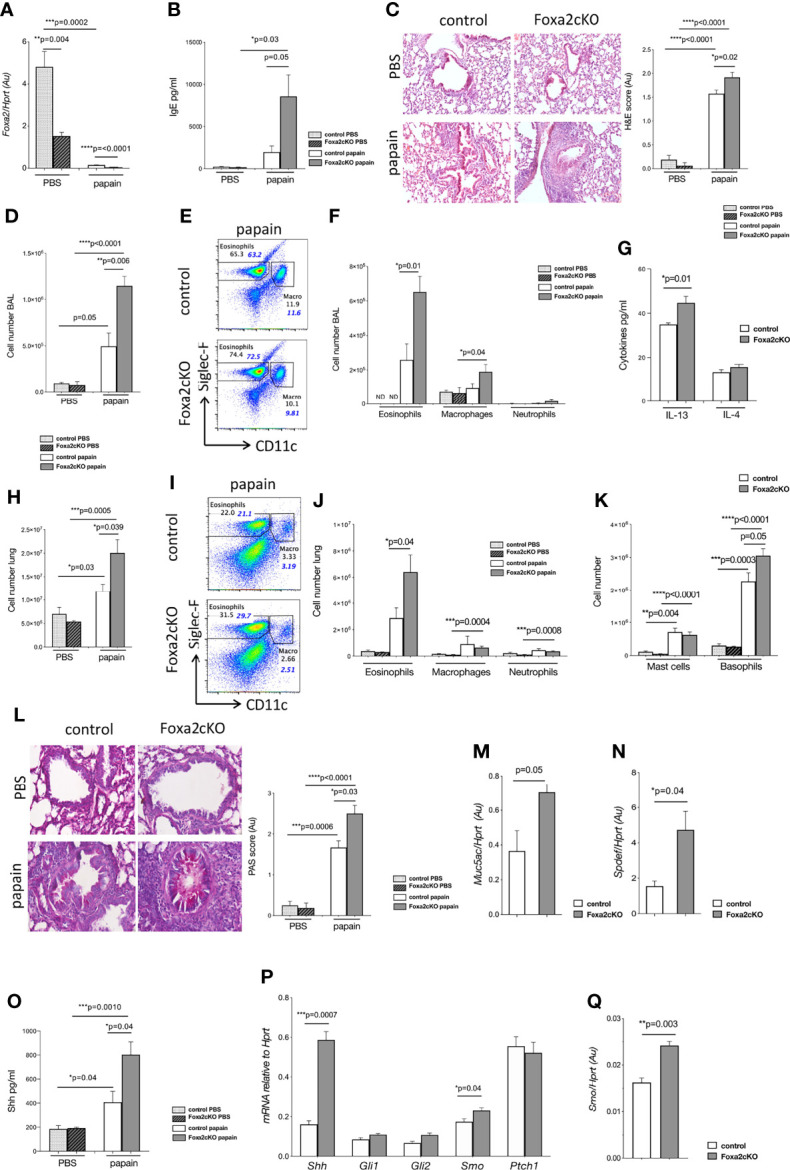
Conditional deletion of Foxa2 from T-cells increases papain-induced allergic airway inflammation. Control and Foxa2cKO mice were immunized intranasally with 25μg of papain or PBS as control at day 0 and day 7 and analysed at day 10 (**Figure S1A**). **(A-C)** Control PBS (n=4); control papain (n=5); Foxa2cKO PBS (n=7); Foxa2cKO papain (n=7). **(A)** *Foxa2* expression (QRT-PCR relative to *Hprt*) in lung homogenates of control and Foxa2cKO mice. **(B)** Total serum IgE concentration (ELISA). **(C)** Representative images showing cellular infiltration in H&E-stained lung sections (20×Objective lens (Plan-Neofluar/0.5NA)). Barchart: score of cellular infiltration of airways, from 0-1 minimal, 1-2 moderate, 2-3 severe infiltration. **(D)** Barchart: number of cells in BAL from control and Foxa2cKO mice after PBS (n=4-5) and papain (n=5-6) treatment. **(E)** Flow cytometry: eosinophils (Siglec-F+CD11c-) and macrophages (Siglec-F+CD11c+) from control and Foxa2cKO BAL after papain-treatment **(F)** Barchart: number of eosinophils (Siglec-F+CD11c-), macrophages (SiglecF+CD11c+) and neutrophils (CD11c-SiglecF-CD11b+Ly6G+) in BAL from control and Foxa2cKO mice after PBS (n=4-5) and papain (n=5-6) treatment. **(G)** Barchart: IL-13 and IL-4 concentration (ELISA) in BAL from control (n=5) and Foxa2cKO (n=5) after papain-treatment. **(H)** Barchart: number of cells in lung from control (n=5) and Foxa2cKO (n=7) groups. **(I)** Flow cytometry: eosinophils (Siglec-F+CD11c-) and macrophages (Siglec-F+CD11c+) from lung after papain-treatment. **(J)** Barchart: number of eosinophils (Siglec-F+CD11c-), macrophages (SiglecF+CD11c+) and neutrophils (CD11c-SiglecF-CD11b+Ly6G+) recovered from control (n=5) and Foxa2cKO (n=7) lung. **(K)** Barcharts: number of mast cells (ckit+FcεRI+DX5-) and basophils (DX5+FcεRI+ckit-) in lung after PBS (n=4-6) and papain treatment (n=5-7). **(L)** PAS staining of lung of control and Foxa2cKO mice from PBS-control (n=4) and papain-control (n=3), PBS-Foxa2cKO (n=4) and papain-Foxa2cKO (n=4) groups. Histology pictures show representative PAS staining of lung sections from the four groups (40x Objective lens (Plan-Neofluar/0.75NA)). Barchart: PAS score, following blind semiquantitative histological assessment to score mucus production: 0–1, minimal; 1–2, moderate; and 2–3, severe. **(M, N)** QRT-PCR for **(M)** *Muc5a*c **(N)** *Spdef* expression in sorted CD45-CD326+ cells from control (n=3) and Foxa2cKO (n=3) lung after papain-treatment. **(O)** Shh (ELISA) of lung homogenate after PBS (n=4) and papain-treatment (n=3) in the different genotypes. **(P)** QRT-PCR of Hh pathway components in sorted CD45-CD326+ cells of control (n=3) and Foxa2cKO (n=3) lung after papain-treatment. **(Q)** *Smo* expression in sorted CD4+CD3+ cells from control (n=3) and Foxa2cKO (n=3) lung after papain-treatment. Non-italicised numbers on plots give percentage of cells in the regions shown (of the gate used to make the plots), italicized numbers in blue indicate percentage of each population out of the total number of cells recovered. Barcharts: mean ± SEM; Au, arbitrary units; ND, not detected; *p<0.05, **p<0.01, ***p<0.001, ****p<0.0001; unpaired Student’s t-test.

We measured *Foxa2* expression in lung tissue from the four groups of mice by QRT-PCR. *Foxa2* expression was lower in PBS-treated Foxa2cKO lung than PBS-treated control, indicating that *Foxa2* transcripts expressed in lung-resident T-cells contribute to the overall levels of *Foxa2* detected in lung under physiological conditions (**Figure 1A**). Expression of *Foxa2* in lung decreased after papain-treatment (**Figure 1A**), consistent with previous studies showing that *Foxa2* is downregulated in lung on induction of Th2 inflammation (7, 12, 14). In the papain-treated Foxa2cKO lung *Foxa2* expression was significantly lower than in papain-treated control, suggesting that a proportion of the *Foxa2* transcripts detected in the control lung on induction of AAD came from T-cells.

We compared total serum IgE between groups to confirm that papain-treatment had led to an allergic response. Papain-treatment led to elevated serum IgE and papain-treated Foxa2cKO mice showed on average >4-fold higher serum IgE compared to papain-treated controls (~8000 pg/ml compared to ~2000 pg/ml), indicating an increased allergic response when Foxa2 was conditionally deleted from T-cells (**Figure 1B**). Histological analysis of lung tissue showed that papain-treatment led to cellular infiltration to lungs, which was significantly higher in the Foxa2cKO than control group (**Figure 1C**).

To evaluate the effect of the allergen on inflammatory cell recruitment we analysed immune cell infiltration into airways. When treated with PBS alone there were no significant differences in the number of cells isolated from BAL or its cellular composition between Foxa2cKO compared to control. Papain-treatment led to an increase in the number of inflammatory cells in the BAL (**Figures 1D–F**). The overall number of cells and the number of eosinophils in BAL were higher in the Foxa2cKO group compared to control after papain-treatment (**Figures 1D–F**). We did not detect a difference in the number of macrophages and neutrophils in BAL between papain-treated Foxa2cKO and control groups, although papain-treatment increased the number of macrophages in papain-treated Foxa2cKO compared to PBS-treated mice (**Figure 1F**). Papain-treatment induced a modestly higher concentration of IL-13 but not IL-4, in BAL from the Foxa2cKO group than controls (on average ~46 pg/ml IL-13 in Foxa2cKO versus 34 pg/ml in control) (**Figure 1G**).

On analysis of lung, we recovered similar numbers of cells from control and Foxa2cKO mice treated with PBS alone (**Figure 1H**). Papain-treatment led to cellular infiltration to the lung, which was increased in the Foxa2cKO mice compared to the control group (**Figures 1H–K**), with ~3-fold higher recruitment of eosinophils (**Figures 1I, J**). The number of basophils was also higher in the lungs of Foxa2cKO mice compared to control after papain-treatment (**Figure 1K**). Papain treatment increased the number of macrophages, neutrophils and mast cells isolated from the lung of Foxa2cKO mice compared to Foxa2cKO PBS-treated lung, but no differences were found in the number of macrophages, neutrophils and mast cells between the two genotypes after papain-treatment (**Figures 1J, K**).

PAS staining showed that mucus-secreting cells were more prevalent in the airway epithelium of Foxa2cKO than control mice after papain-treatment (**Figure 1L**). Likewise, expression of the mucin gene *Muc5ac* and its transcriptional activator *Spdef*, were significantly higher in FACS-sorted lung epithelial cells from Foxa2cKO mice compared to controls after papain-treatment, consistent with increased mucus production (**Figures 1M, N**).

Shh protein concentration was increased in lung after papain-treatment, and was ~2-fold higher in Foxa2cKO lung than control (~800 pg/ml compared to ~400 pg/ml; **Figure 1O**). QRT-PCR analysis of FACS-sorted lung epithelial cells confirmed higher expression of *Shh* and its signal transduction molecule *Smoothened* (*Smo*) in Foxa2cKO compared with control after papain-treatment (**Figure 1P**). Expression of *Smo* was also significantly higher in FACS-sorted lung CD4 T-cells from Foxa2cKO group compared to controls after papain-treatment (**Figure 1Q**), consistent with the higher concentration of Shh in the lung (**Figure 1O**).

### Foxa2 Deficiency Enhances the Th2 Immune Response Upon Papain Treatment

**Figure 2:**
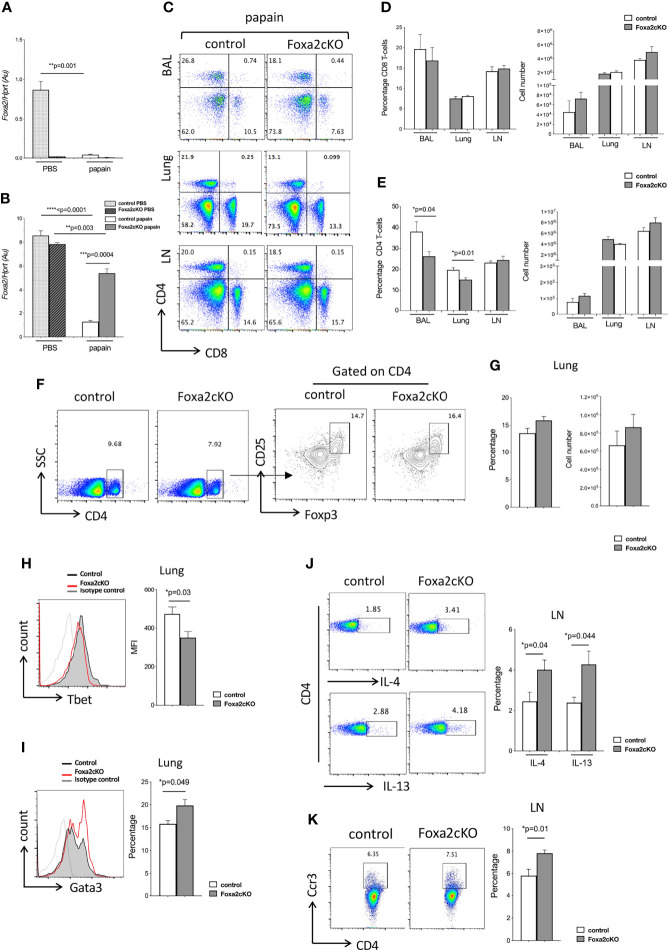
Absence of Foxa2 in T-cells enhances Th2-mediated disease in AAD. Control and Foxa2cKO mice were immunized intranasally with 25μg papain (or PBS as control) at day 0 and day 7 and analysed at day 10. **(A, B)** *Foxa2* expression (QRT-PCR) representative of three independent experiments of sorted **(A)** CD4+CD3+ T-cells and **(B)** CD45-CD326+ epithelial cells from lungs of control (n=3) and Foxa2cKO (n=3) groups after PBS and papain-treatment. **(C)** Flow cytometry: T-cell populations in control and Foxa2cKO BAL, lung and mLN after papain-treatment. **(D, E)** Barcharts show percentage and cell number of **(D)** CD8 and **(E)** CD4 T-cells in BAL, lung and mLN from papain-treated control and Foxa2cKO mice (n=4-7). **(F, G)** Flow cytometry (lung) from control (n=3) and Foxa2cKO (n=3) after papain-treatment. **(F)** Dot plots: CD4 expression versus SSC. Contour plots: CD25 and icFoxp3 expression gated on lung CD4+ cells. **(G)** Barcharts: percentage (left) of CD25+icFoxp3+ cells in CD4+ population; number (right) of CD4+CD25+icFoxp3+ cells. **(H)** Histogram: icTbet expression in CD4 T-cells in control and Foxa2cKO lungs after papain-treatment. Barchart: mean fluorescence intensity (MFI) of icTbet in CD4 T-cells from control and Foxa2cKO lungs after papain-treatment. **(I)** Histogram: icGata3 expression from control and Foxa2cKO lungs after papain-treatment. Barchart: percentage of icGata3+ cells in lung CD4 T-cells from control and Foxa2cKO after papain-treatment. For **(H, I)**, grey histograms show isotype control antibody staining; control (n=4) and Foxa2cKO (n=6). **(J)** Flow cytometry: icIL-4 (upper) and icIL-13 (lower) in CD4 T-cells from control and Foxa2cKO mLN after papain-treatment. Barchart: percentage of cytokine-positive cells from control (n=5) and Foxa2cKO mice (n=7) after papain-treatment. **(K)** Flow cytometry: Ccr3 expression on CD4 T-cells from control and Foxa2cKO mLN after papain-treatment. Barchart: percentage of Ccr3+ cells in CD4 T-cell population in mLN from control (n=5) and Foxa2cKO (n=7) after papain-treatment. Au: arbitrary units; Barcharts: mean ± SEM; *p<0.05; **p<0.01; ***p<0.001, ****p<0.0001, unpaired Student’s t-test.

Overall, conditional deletion of Foxa2 from T-cells resulted in measurably more severe AAD on papain treatment, with an increase in total serum IgE; IL-13 in BAL; inflammatory cell infiltration to lung and BAL; mucus production; and Shh expression, suggesting that, as in lung epithelial cells, in lung T-cells Foxa2 acts to reduce AAD. In support of this, *Foxa2* expression was lower in FACS-sorted lung CD4 T-cells from papain-treated control mice than in their PBS-treated counterparts (**Figure 2A**). Expression of *Foxa2* in FACS-sorted lung epithelial cells also decreased after papain-treatment (**Figure 2B**). However, suprisingly its expression was higher in the Foxa2cKO CD45-CD326+ epithelial cells compared to controls after papain-treatment.

We compared recruitment of T-cells after papain-treatment in BAL, lung and mLN in Foxa2cKO mice compared to control (**Figures 2C–E**). We found no differences in the proportion of CD8 T-cells between genotypes, but the percentage of CD4 T-cells isolated from BAL and lung were significantly lower in the Foxa2cKO compared to control after papain-treatment (**Figures 2C–E**). However, given the increased number of cells isolated overall, the number of CD4 and CD8 T-cells isolated from BAL and lung were not significantly different between the two genotypes (**Figure 2E**). As regulatory T-cells (Treg) may suppress inflammation in allergic asthma (46), we analysed the lung Treg population. We found no difference in the number or proportion of CD4+CD25+Foxp3+ cells isolated after papain-treatment from lung of control and Foxa2cKO mice (**Figures 2F, G**), suggesting that a reduction in the Treg population did not account for the more severe AAD observed in the Foxa2cKO lung. However, when we assessed the expression of Th1 and Th2 transcription factors in lung CD4 T-cells after papain-treatment we found that expression (mean fluorescence intensity) of the hallmark Th1 transcription factor Tbet was significantly lower on CD4 T-cells from Foxa2cKO mice compared to control (**Figure 2H**), whereas the proportion of lung CD4 T-cells that expressed the key Th2 transcription factor Gata3 was higher (on average ~15% in control compared to ~20% in Foxa2cKO), indicative of an increase in the Th2 effector population (**Figure 2I**).

The percentage of CD4 T-cells that expressed icIL-4 and icIL-13 in mLN were ~2-fold higher in Foxa2cKO mice than control after papain-treatment (on average ~2% in control and ~4% in Foxa2coKO for each cytokine; **Figure 2J**). The proportion of mLN CD4 T-cells that expressed Ccr3, a chemokine receptor that signals to attract eosinophils and basophils and is predominately expressed in Th2 cells (47) was also significantly but modestly higher in Foxa2cKO mice compared to controls after papain-treatment, rising from on average ~5.8% in the control to ~7.9% in the Foxa2cKO (**Figure 2K**).

Taken together, these data indicated that Foxa2 deficiency in T-cells is associated with Th2-mediated disease and that it may act in T-cells, as well as in respiratory epithelial cells to reduce/prevent Th2-mediated AAD.

### Conditional Deletion of Foxa2 Promotes Th2 Differentiation *In Vitro*

**Figure 3:**
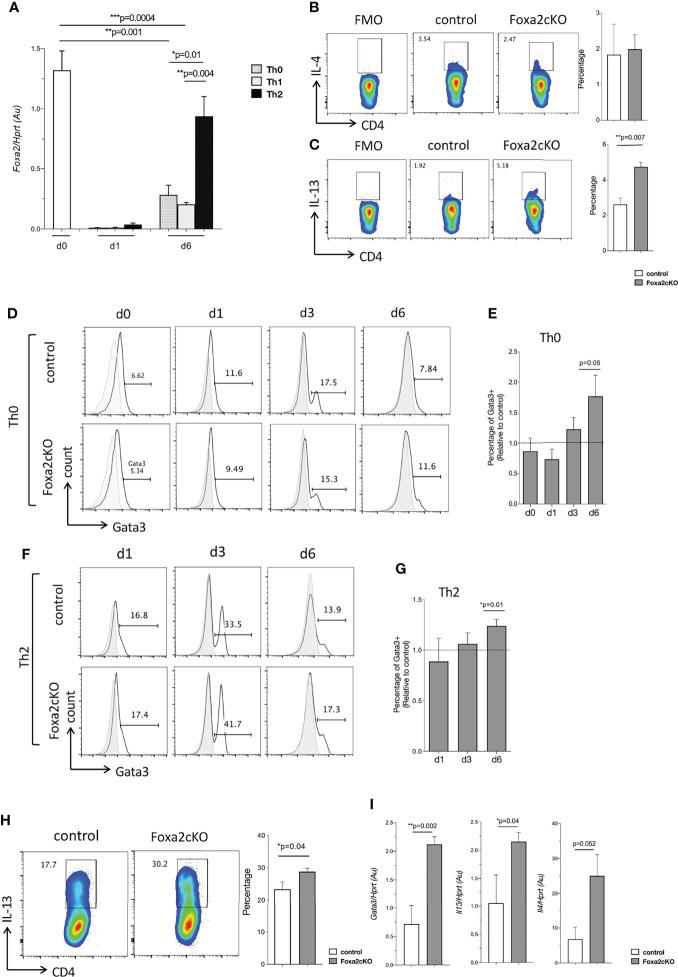
Effect of conditional deletion of Foxa2 from T-cells on Th2 differentiation *in vitro*. **(A)** Barchart: *Foxa2* expression (QRT-PCR) in purified unstimulated WT CD4 T-cells and CD4 T-cells activated with anti-CD3/anti-CD28, cultured in Th0, Th1 and Th2 conditions at 1 day (d1) and day 6 (d6). QRT-PCR values pooled from 2 independent experiments with biological replicates. **(B, C)** Flow cytometry: **(B)** icIL-4 and **(C)** icIL-13, in CD4 T-cells isolated from control and Foxa2cKO spleen and stimulated for 4 hours with Ionomycin, PMA, and Brefeldin A. FMO: Fluorescence minus one as negative control for cytokine staining. Barcharts: percentage iccytokine-positive cells in control (n=3) and Foxa2cKO CD4 T-cells (n=3). **(D-H)** Purified CD4 T-cells from control (n=5) and Foxa2cKO (n=6) mice activated in Th0 and Th2 conditions and analysed at different time points. **(D)** Flow cytometry: icGata3 expression in CD4 T-cells in Th0 conditions at (unstimulated, day (d) 0) and on day 1, 3 and 6 from control and Foxa2cKO mice, giving percentage of cells in the marker shown. **(E)** Barchart: percentage of icGata3+ CD4 T-cells relative to controls in Th0 conditions. **(F)** Flow cytometry analysis of icGata3 expression in CD4 T-cells in Th2 conditions at day (d) 1, 3 and 6 from control and Foxa2cKO mice, giving percentage of cells in the marker shown. **(G)** Barchart: percentage of icGata3 cells relative to control cultures under Th2 conditions. **(D, F)** Grey histograms show isotype control antibody staining. **(H)** Flow cytometry: icIL-13 expression in Th2 conditions in purified CD4 T-cells from control and Foxa2cKO mice cultured for 6 days, following 4 hours restimulation (in presence of Brefeldin A). Barchart: percentage of icIL-13+ cells in Th2 conditions from control (n=5) and Foxa2cKO mice (n=6). **(I)** Barcharts: *Il13*, *Il4* and *Gata3* expression (QRT-PCR) in purified CD4 T-cells from control and Foxa2cKO mice after 6 days culture in Th2 conditions and 16h restimulation with antiCD3/CD28; QRT-PCR values pooled from two independent experiments with biological replicates. Au: arbitrary units. Barcharts: mean ± SEM; *p < 0.05, **p < 0.01, ***p < 0.001, unpaired Student’s t-test.

To investigate the cellular mechanism underlying the inhibition of AAD by Foxa2 in T-cells we tested if Foxa2 regulates T helper differentiation. We carried out *in vitro* polarisation experiments in which purified CD4 T-cells were activated with anti-CD3/anti-CD28 under Th-skewing conditions: Th0 (absence of differentiating cytokines), Th1 (rIL-12, anti-IL-4 mab) and Th2 (rIL-4, anti-IL-12 mab, anti-IFNγ mab). To test if *Foxa2* expression was influenced by T-cell activation and differentiation signals we first compared *Foxa2* expression in WT purified CD4 T-cells (unstimulated) and WT CD4 T-cells activated in skewing conditions. *Foxa2* expression was downregulated after one day of activation. After 6 days, *Foxa2* expression had increased, with >2-fold higher expression in Th2 cultures compared with Th0 and Th1 (**Figure 3A**).

We then tested if fresh unstimulated Foxa2-deficient CD4 T-cells showed inherent bias toward Th2 differentiation, by staining splenocytes for icTh2 cytokine expression after 4 hours’ stimulation in presence of Brefeldin A. No differences were found in the percentage of cells that stained positive with anti-IL-4 between Foxa2cKO and control CD4 T-cells (**Figure 3B**), but interestingly the percentage that stained positive for icIL-13 was ~2-fold higher in Foxa2cKO compared to control (1.92% in control compared to 5.18% in Foxa2cKO), suggestive of a possible bias towards Th2 (**Figure 3C**).

Since more Foxa2 deficient CD4 T-cells showed IL-13 cytokine expression *ex vivo* and WT Th2 cultures expressed higher levels of *Foxa2*, we determined the effect of Foxa2-deficiency on Th1/Th2 polarization. We stimulated purified CD4 T-cells from control and Foxa2cKO mice under Th0, Th1 and Th2-skewing conditions, and analysed by flow cytometry at day 1, 3 and 6 of culture, using Tbet and Gata3 expression as markers of Th1 and Th2 identity respectively. At the start of the experiment (unstimulated, day 0) and when cultured under Th0 conditions for 1 and 3 days, the percentage of Gata3+ cells were not different between control and Foxa2cKO cells, whereas on day 6, 11.6% of Foxa2cKO CD4 T-cells expressed Gata3 compared to 7.8% in control (**Figures 3D, E**). Under Th2 culture conditions, there were again no significant differences between Foxa2cKO and control on day 1 and 3, but on day 6 a greater proportion of Foxa2cKO CD4 T-cells expressed Gata3 than their control counterparts (17.3% in Foxa2cKO compared to 13.9% in control; **Figures 3F, G**), suggesting that differentiation to the Th2 effector subset took place more efficiently when Foxa2 was conditionally deleted from T-cells. In support of this, at the end of the culture period 30.2% CD4 T-cells expressed icIL-13 in Foxa2cKO Th2 cultures compared to 17.7% in control Th2 cultures (**Figure 3H**). QRT-PCR confirmed that expression of *Gata3, Il13* and *Il4* were higher in the Foxa2cKO Th2 cultures than in control Th2 cultures (**Figure 3I**).

**Figure 4:**
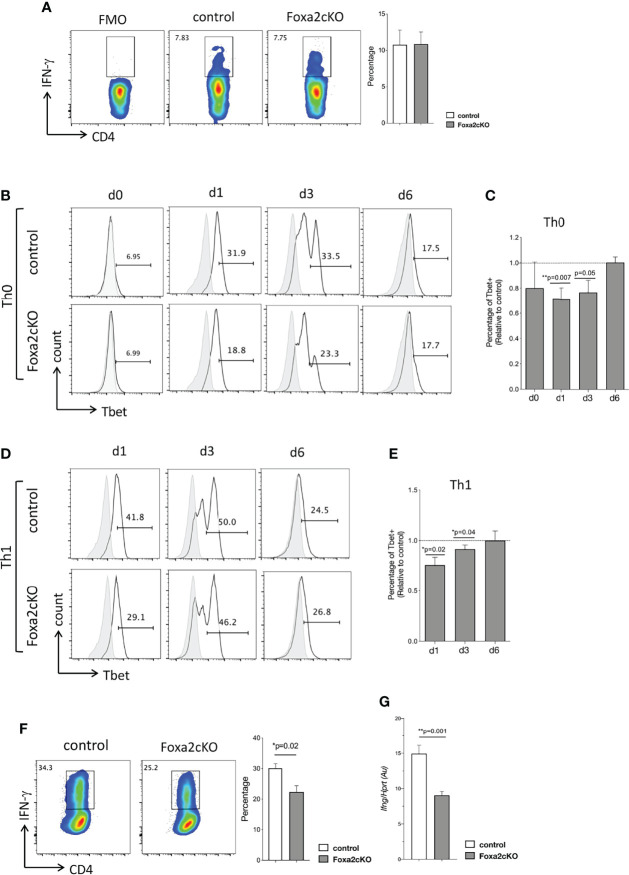
Absence of Foxa2 from T-cells reduces Th1 differentiation *in vitro*. **(A)** Flow cytometry: icIFN-γ expression in CD4 T-cells from control and Foxa2cKO mice spleen, after 4 hours’ stimulation with Ionomycin, PMA, and Brefeldin A. Barchart: percentage icIFN-γ+ cells in control (n=3) and Foxa2cKO CD4 T-cells (n=3). **(B–F)** Purified CD4 T-cells from control (n=5) and Foxa2cKO (n=6) mice stimulated in Th0 and Th1 conditions. **(B)** Flow cytometry: icTbet expression in CD4 T-cells in Th0 conditions at the start of the experiment (unstimulated, day **(d)** 0) and on day 1, 3 and 6, giving percentage of cells in the marker shown. **(C)** Barchart: percentage of icTbet+ cells relative to control cultures in Th0 conditions. **(D)** Flow cytometry: icTbet expression in CD4 T-cells in Th1 conditions at day 1, 3 and 6, giving percentage of cells in the marker shown. **(B, D)** Grey histograms show isotype control antibody staining. **(E)** Barchart: percentage of icTbet+ cells relative to control cultures after 6 days in Th1 conditions. **(F)** Flow cytometry: icIFN-γ expression in purified control and Foxa2cKO CD4 T-cells after 6 days’ culture in Th1 conditions, following 4 hour restimulation (in presence of Brefeldin A). Barchart: percentage of icIFN-γ+ cells in CD4 T-cells cultured in Th1 conditions for 6 days (n=4). **(G)** *Ifng* expression (QRT-PCR) in purified control and Foxa2cKO CD4 T-cells after 6 days of culture in Th1 conditions and 16h of restimulation with anti-CD3/anti-CD28; QRT-PCR values pooled from two independent experiments with biological replicates. Au: arbitrary units. Barcharts: mean ± SEM; *p< 0.05; **p<0.01; unpaired Student’s t-test.

We next compared the ability of Foxa2cKO and control CD4 T-cells to differentiate to the Th1 effector subset *in vitro*. At the start of the experiment, we did not detect a difference in icIFN-γ or icTbet expression between Foxa2cKO and control CD4 T-cells (**Figures 4A–C**). However, when cultured in Th0 conditions the proportion of Tbet+ cells was significantly lower in Foxa2cKO CD4 T-cells compared to control on day 1 (18.8% in Foxa2cKO compared to 31.9% in control) and day 3 (23.3% in Foxa2cKO compared to 33.5% in control) (**Figures 4B, C**). After 6 days the percentage of Tbet+ cells had reduced from its peak on day 3 and was no longer different between genotypes (17.7% in Foxa2cKO compared to 17.5% in control) (**Figures 4B, C**). Likewise, when cultured in Th1 conditions, the proportion of Tbet+ cells was significantly lower in Foxa2cKO CD4 T-cells compared to control on day 1 (29.1% in Foxa2cKO compared to 41.8% in control) and day 3 (46.2% in Foxa2cKO compared to 50.0% in control) (**Figures 4D, E**). After 6 days the percentage of Tbet+ cells had reduced from its peak on day 3 and was no longer different between genotypes (26.8% in Foxa2cKO compared to 24.5% in control) (**Figures 4D, E**). However, on day 6, 25.2% CD4 T-cells expressed IFN-γ in the Foxa2cKO Th1 cultures compared to 34.3% in their control counterparts (**Figure 4F**) and QRT-PCR confirmed that expression of *Ifng* was lower in Foxa2cKO Th1 cultures than control (**Figure 4G**). Taken together, these data show that Foxa2 is involved in Th1/2 polarization. It promotes efficient differentiation to the Th1 effector subset *in vitro* and in its absence Th2 differentiation is increased.

## Discussion

Low Foxa2 expression in lung is associated with Th2 inflammation and cytokine production, and over-production of mucus in humans and mice (7, 12, 14, 48). Here, we found that *Foxa2* expression was downregulated in lung tissue and control lung CD4 T-cells on induction of AAD by papain sensitisation. Conditional deletion of Foxa2 from T-cells led to more severe AAD, indicating that Foxa2 expression in T-cells is important for physiological lung homeostasis, and suggesting that Foxa2 activity broadly prevents Th2-inflammation, when expressed in either T-cells or in lung epithelial cells.

To investigate the cellular mechanism of the increase in AAD severity in Foxa2cKO mice, we tested if Foxa2-deficiency directly altered Th1/Th2 differentiation. Fresh resting Foxa2-deficient CD4 T-cells isolated from spleen did not show obvious bias towards Th2 polarisation, but interestingly this population did contain an increased proportion of icIL-13+ cells. When activated *in vitro*, however, Foxa2-deficient CD4 T-cells differentiated less efficiently to the Th1 effector subset in Th0 and Th1 conditions than controls, and in contrast, their Th2 differentiation was increased in Th0 and Th2 conditions, and the Foxa2-deficient cultures expressed more *Il13*, *Il4* and *Gata3*. Taken together, these experiments suggest that T-cell intrinsic bias towards Th2 differentiation directly caused more severe AAD in Foxa2cKO mice, rather than the increased disease severity arising because of a reduction in immune regulation by CD4+ Tregs or a change in function of another immune cell population.

Th1 and Th2 differentiation are antagonistic processes, in which lineage-specific cytokine signalling and transcription factors mutually suppress one another’s activities to reinforce differentiation to one effector population over another (49). Tbet suppresses transcription of *Gata3* and also inhibits Gata3 function through direct protein-protein interaction (50–52); whereas Gata3 represses *Stat4* and inhibits IFN-γ production (53, 54). We found that Foxa2 was rapidly downregulated on T-cell activation *in vitro* and more highly expressed in Th2 cells than Th1 cells after 6 days in culture, but promoted efficient Th1 differentiation, suggesting that Foxa2 might be involved early after activation in the regulation of Th1/2 polarisation to prevent Th2 differentiation and/or enable Th1 differentiation; and that Foxa2 serves to limit the Th2 response in T-cells, as has been described previously in lung epithelial cells (7, 14). In our experiments it seems likely that failure of Foxa2-deficient CD4 T-cells to upregulate Tbet fully early on activation led to default adoption of a Th2 fate, leaving Gata3 less inhibited to repress Th1 cytokine pathways.

In addition to its ability to control transcription of its target genes directly, Foxa2 can function as a pioneer transcription factor, which binds silent condensed chromatin early in a developmental programme to open up local chromatin to allow other transcriptional activators to bind; and it has been shown to demethylate specific regions of DNA to generate stable lineage-specific DNA methylation patterns; and to regulate RNA splicing (33, 55, 56). The way in which Foxa2 influences Th1/2 differentiation in T-cells is unknown and will require further research to investigate its impact on chromatin conformation, DNA methylation, RNA processing and transcription.

In lung epithelial cells Foxa2-deficiency increases IL-13 expression, whereas IL-13 signalling to WT lung epithelial cells down-regulates Foxa2 (16). In this study, we observed that Foxa2-deficiency in T-cells also increased their IL-13 expression, but unexpectedly, *Foxa2* expression in FACS-sorted Foxa2cKO lung epithelial cells was not down-regulated by papain-treatment to the same degree as in control papain-treated epithelial cells. This finding was surprising, given the generally increased severity of AAD, increased mucus production and IL-13 production by T-cells observed in the Foxa2cKO compared to control, and it is in contrast to many studies which have shown that Foxa2 down-regulation leads to increased mucus production on induction of AAD. The reasons for this discrepancy are unclear, but may be because of the heterogeneity of the respiratory epithelial cell population. Alternatively, it may be because additional signals provided by immune-cells (for example EGFR signalling), are required for *Foxa2* downregulation in epithelial cells, and these signals may be dependent on immune-cell expression of Foxa2.

Increased Shh expression and Hh signalling to T-cells promote AAD (23, 24, 28, 29) and Foxa2 is associated with the Hh signalling pathway in T-cells: the *Foxa2* promoter contains binding sites for the Hh-responsive transcription factors Gli1 and Gli2, and *Foxa2* is a Shh target gene in early T-cell development (19, 32). Our study suggests that Hh pathway activation and Foxa2 have opposing functions in lung T-cells, as Foxa2-deficiency increased Th2 differentiation, AAD severity and *Smo* expression, whereas Shh signalling promotes Th2 differentiation and allergic asthma. In support of this, Foxa2 and Shh signalling have opposite functions in positive selection during T-cell development, where Hh pathway activation inhibits positive selection, but Foxa2 is required for positive selection (33, 57–60). The relationship between Foxa2 activity and Hh signalling in T-cells will require more investigation and it will be important to investigate if *Foxa2* is a Hh target gene and negative regulator of Hh signalling in lung T-cells. As FOXA2 is expressed in human CD4 T-cells (GSE179609; (61), it will also be important to investigate the function of FOXA2 in T-cells in allergic asthma in people.

In summary, our study showed that Foxa2 influences T helper effector differentiation. Conditional deletion of Foxa2 from T-cells increased Th2 differentiation *in vitro* and increased disease severity of AAD.

## Data Availability Statement

The original contributions presented in the study are included in the article/**Supplementary Material**. Further inquiries can be directed to the corresponding author.

## Ethics Statement

The animal study was reviewed and approved by UCL ethics committee.

## Author Contributions

DY and TC conceived experiments and wrote the paper. DY, C-IL, and AF designed and performed experiments. EP, MC, JR, and SR performed experiments. All authors critically reviewed the manuscript. All authors contributed to the article and approved the submitted version.

## Funding

This work was supported by grants from the MRC, BBSRC and GOSHCC.

## Conflict of Interest

The authors declare that the research was conducted in the absence of any commercial or financial relationships that could be construed as a potential conflict of interest.

## Publisher’s Note

All claims expressed in this article are solely those of the authors and do not necessarily represent those of their affiliated organizations, or those of the publisher, the editors and the reviewers. Any product that may be evaluated in this article, or claim that may be made by its manufacturer, is not guaranteed or endorsed by the publisher.

## Supplementary Material

Supplementary Figure 1
**(A)** Cartoon illustrates the papain-protocol: WT mice were immunized (intranasally i.n) with 25μg of papain or PBS as control to induce allergic airway inflammation. **(B-E)** WT mice were immunized intranasally with 25μg of papain (n=6) or PBS as control (n=5) at day 0 and day 7 and analysed at day 10. Flow cytometry plots illustrate gating strategies for this allergic airway disease model. **(B)** BAL was gated to exclude debris using forward (FSC) and side (SSC) scatter. Singlets were selected using FSC-A against FSC-H. Single cells were analysed to identify eosinophils (Siglec-F+CD11c-), macrophages (Siglec-F+CD11c+), and neutrophils (Siglec-F-CD11c-Ly-6G+CD11b+). Bar charts: number of the cells recovered from BAL, and number of eosinophils, macrophages and neutrophils recovered from BAL. **(C)** Cell suspension from lung was gated to exclude debris using FSC against SSC. Singlets were selected using FSC-A against FSC-H. Single cells were analysed to identify eosinophils (Siglec-F+CD11c-), macrophages (Siglec-F+CD11c+), and neutrophils (Siglec-F-CD11c-Ly-6G+CD11b+). Barcharts: number of cells in lung, and number of eosinophils, macrophages and neutrophils in lung. **(D)** Flow cytometry analysis of lung basophils (DX5+FcεRI+ckit-), gated to be DX5+FcεRI+ckit- and then by FSCvsSSC profile. Bar chart: number of basophils recovered from lung. **(E)** Flow cytometry analysis of lung mast cells (ckit+FcεRI+DX5-), gated to be ckit+FcεRI+DX5-, and then by FSCvsSSC profile. Barcharts: number of basophils recovered from lung. Non-italicized numbers give percentage of cells in the region of the plot shown, as a percentage of the gates used to make the plot. Numbers in italics in blue indicate the percentage of each population from the total number of cells recovered. ND: not detected. Barcharts: mean ± SEM; *p<0.05; **p<0.01; ***p<0.001, ****p<0.0001, unpaired Student’s t-test.

Supplementary Figure 2 Further analyses of WT mice immunized intranasally with 25μg of papain or PBS as control at day 0 and day 7 and analysed at day 10. **(A-C)** Barcharts: number of CD4 T-cells recovered from **(A)** BAL, **(B)** lung and **(C)** LN from PBS (n=5) and papain (n=6) groups. **(D)** Total serum IgE concentration (ELISA) for PBS (n=4) and papain (n=4) groups. **(E, F)** Barchart: IL-13 and IL-4 concentration (ELISA) in **(E)** BAL and **(F)** lung from PBS (n=3) and papain (n=3). **(G)** *Il13 and Il4* expression (QRT-PCR relative to *Hprt*) in lung homogenates of PBS (n=2) and papain (n=2). **(H)** *Il13 and Il4* expression (QRT-PCR relative to *Hprt*) in sorted CD4+CD3+ cells from PBS (n=2) and papain (n=2). Barcharts: mean ± SEM; Au: arbitrary units; *p<0.05, **p<0.01, ****p<0.0001; unpaired Student’s t-test.

Supplementary Figure 3
**(A**) *Foxa2* expression in FACS-sorted CD45-CD326+, CD4+CD3+, CD8+CD3+ and CD4+CD11c+ isolated from lungs of control (n=3) and Foxa2cKO (n=3). Barcharts: mean ± SEM; **p<0.01.
