# Supplementary material for: The Pioneer Transcription Factor Foxa2 Modulates T Helper Differentiation to Reduce Mouse Allergic Airway Disease
Source: Front Immunol. 2022 Aug 8;13:890781. doi: 10.3389/fimmu.2022.890781 (PMC9393229; doi:10.3389/fimmu.2022.890781)
Supplement: Supplementary file 4 [file Table_1.docx]

**Supplementary Table 1**

List of antibodies and cytokines used in these experiments, giving clone name, fluorochrome, catalog number and supplier.
